# Immune responses and recovery from spotty liver disease in layer birds

**DOI:** 10.1016/j.psj.2025.105351

**Published:** 2025-05-26

**Authors:** Sarah Eastwood, Timothy B. Wilson, Jiongrui Huang, Bronwyn E. Campbell, Peter C. Scott, Robert J. Moore, Thi Thu Hao Van

**Affiliations:** a School of Science, RMIT University, Bundoora West Campus, Bundoora, Victoria, Australia; b Scolexia Pty Ltd., Moonee Ponds, Victoria, Australia

**Keywords:** Spotty liver disease, *Campylobacter hepaticus*, Reinfection, Immune response, Gene expression

## Abstract

Spotty Liver Disease (SLD), caused by *Campylobacter hepaticus*, greatly impacts the health and egg production of affected layer hens and is a disease of concern in the poultry industry. This study aimed to enhance the understanding of the immune response in chickens to *C. hepaticus* infection and their ability to resist reinfections. One hundred- and twenty-layer chickens were allocated to 10 groups and challenged, from one to three times, with *C. hepaticus* HV10^T^, with six weeks between each challenge. Blood and cloacal swabs were collected every three weeks to assess antibody responses and *Campylobacter* presence. Upon necropsy, bile, spleen, jejunum, and blood samples were collected for *C. hepaticus* detection and host gene expression analysis using qPCR and RNA sequencing. We found that most birds challenged with *C. hepaticus* for the second or third time did not develop liver lesions even with the presence of *C. hepaticus* in their bile, suggesting that birds were resistant to disease development following repeated exposure. Anti-*C. hepaticus* antibodies increased significantly six weeks after a single challenge but reduced from nine weeks. Quantitative PCR demonstrated that *C. hepaticus* could be recovered from the bile six weeks after a single challenge and increased significantly after a secondary challenge. RNA-seq and qPCR data demonstrate an elevation in pro-inflammatory cytokines, such as interleukin 6 (IL-6) and interleukin 1ß (IL-1ß), after a secondary challenge and down-regulation during a third challenge. Expression of many genes encoding barrier-supporting proteins genes were differentially expressed, with increased expression following a third challenge compared to expression after a single challenge. Comparison of gene expression in tissues of triple to a single challenged birds demonstrated that many genes involved in cytokine activity and the JAK-STAT cascade were down-regulated whereas other immune system pathways were up-regulated. Altogether, the results indicate that over time, immune memory, enhanced barrier function, and a balanced immune response developed, resulting in reduced impact of infection on the birds. These findings show that the impact of *C. hepaticus* infection can be ameliorated by immune responses and hence indicate that vaccines that induce appropriate protective immune responses should provide an effective tool to reduce SLD in poultry.

## Introduction

Spotty Liver Disease (SLD) presents one of the most pressing challenges for the poultry industry, especially among extensively housed long lived poultry, including free-range and barn egg layer flocks. The disease is characterised by the formation of whitish grey miliary parenchymal lesions on the liver, an increase of up to 10 % in flock mortality rates and a reduction in egg production of between 10 and 25 % (Crawshaw and Young, 2003; Burch, 2005; Grimes and Reece, 2011). *Campylobacter hepaticus*, and less frequently, *Campylobacter bilis*, have been confirmed as the cause of the disease (Van et al., 2023). Current strategies to reduce the incidence of SLD include maintenance of high-level biosecurity and animal husbandry (Courtice et al., 2018; Grimes and Reece, 2011). Antibiotic treatment, including use of chlortetracycline, amoxycillin, or lincospectin is the primary treatment of SLD, however, efficacy has been compromised by the emergence of antibiotic-resistant strains of *C. hepaticus* in some flocks (Grimes and Reece, 2011; Scott, 2016). The addition of phytobiotics to feed has aided in protection and autogenous vaccines have been used in the field with variable success rates (Scott et al., 2021; Quinteros et al., 2021). Our field observations indicate that flocks can experience repeated outbreaks of SLD. It is not known whether, in second and subsequent outbreaks, all birds in a flock are susceptible to disease development, or if it is only the birds that were not infected in the first outbreak that show disease symptoms in subsequent outbreaks. Therefore, it is important to enhance our understanding of the pathogenesis of SLD, the immune responses of infected birds, and especially their resistance or susceptibility to reinfection.

Cytokines are a group of proteins that play critical roles in regulating immune responses including inflammation and immune cell activation. Pro-inflammatory cytokines such as interleukin 1β (IL-1β), interleukin 6 (IL-6) and interleukin 17a (IL-17a) are produced during stressful periods and regulate the acute phase response (APR) to stress (Kushner, 1993). IL-1β induces the production of IL-6 (Weber et al., 2010) and during tissue damage, APR maintains homeostasis and promotes healing via the activation of innate immunity (Cray et al., 2009). Several studies have reported that acute heat stress (AHS) increased IL-6 production, subsequently inducing tissue protection mechanisms (Leon, 2007; Welc et al., 2012; Phillips et al., 2015). Understanding the role of cytokines in the reinfection of SLD helps evaluate their potential as therapeutic targets for this disease.

Intestinal epithelial cells are tightly connected by intercellular junctional complexes which regulate the movement of molecules and microbes through paracellular pathways and are critical for maintenance of epithelial barrier integrity (Krause et al., 2008). Tight junctions (TJ) are complexes of transmembrane proteins which attach polarized cells to neighbouring cells at the apical region of their lateral membranes (Umeda et al., 2006). The composition of TJ complexes has an important role in establishing the paracellular barrier by maintaining channels that allow or restrict the movement of fluids, electrolytes, macromolecules and luminal microorganisms (Markov et al., 2004). TJ proteins are classified into two classes: barrier-forming or scaffolding and pore-forming types. During an infection, a loss of TJ’s integrity enables migration of pathogens and diffusion of toxins from the gut mucosa into the circulatory system of the host (Tomita et al., 2004; Lamb-Rosteski et al., 2008; Krug et al., 2009; Pinton et al., 2009).

The immune responses associated with SLD in chickens and their responses to re-exposure have yet to be determined. This study aimed to enhance understanding of chicken immune responses to *C. hepaticus* exposure, focusing on their ability to resist and recover from re-exposures, as well as the mechanisms involved.

## Materials and methods

### Animal trial and sample collection

The *C. hepaticus* strain HV10^T^ was cultured as described by Phung et al. (2021). In brief, HV10^T^ was streaked onto horse blood agar (HBA) plates (Brucella broth supplemented with 1.5 % agar and 5 % defibrinated horse blood). *C. hepaticus* was then grown in Brucella broth supplemented with l-cysteine (0.4 mM), l-glutamine (4 mM) and sodium pyruvate (10 mM) in tissue culture T75 flasks at 37°C for 48 h in microaerophilic conditions and used directly for the challenge at various timepoints.

The animal trial was conducted at the Scolexia Animal Research Facility (SCARF, Keilor East, Victoria, Australia). The animal experimentation was approved by the Wildlife and Small Institutions Animal Ethics Committee of the Victorian Department of Economic Development, Jobs, Transport and resources (Project 33.21). One hundred and twenty 19-week-old Hy-Line Brown hens from a farm monitored as free from SLD, and which were negative for *C. hepaticus* and *C. bilis* on PCR assessment of cloacal swabs (Van et al., 2017a, Van et al., 2017b), were used in the trial. Chickens were housed in groups of 3 birds per cage, with 4 cages per group: a total of 12 birds per group (*n* = 12) and 10 groups in total. Unchallenged control birds (Groups 2, 4 and 8) were used as, negative control groups and were orally inoculated with 1 mL of sterile Brucella broth. Challenge control groups (positive control groups) were dosed once with 1 mL of Brucella broth containing 1 × 10^9^ CFU/ml of *C. hepaticus* (Groups 1, 6 and 10). As three *C. hepaticus* challenges were conducted for this trial, three challenged control groups and three unchallenged control groups were required to ensure the challenge model was working at every challenge in birds at different ages. All birds were necropsied six days post-challenge, and birds challenged more than once received *C. hepaticus* at 6-week intervals unless stated otherwise. Birds in Group 5 were challenged once and necropsied 6 weeks later. Birds in Group 3 and 9 were challenged twice, Group 3 at the first and second challenges and Group 9 at the first and third challenges round (a 12-week gap between challenges). Group 7 birds were challenged three times. Table 1 describes the trial design.

**Table 1:** Design of exposure and re-exposure animal trial.

Treatment	Week	Group^1^									
		1	2	3	4	5	6	7	8	9	10
**1st challenge**	0	+	-	+	-	+	-	+	-	+	-
**1st necropsy**	6 d	K^2^	K								
**2nd challenge**	6 wks			+	-	-	+	+	-	-	-
**2nd necropsy**	6 wks 6 d			K	K	K	K				
**3rd challenge**	12 wks							+	-	+	+
**3rd necropsy**	12 wks 6 d							K	K	K	K

1 Groups 2, 4 & 8 = negative controls; Groups 1, 5 & 10 = positive controls for the challenges. Test groups: + = infection with *C. hepaticus* HV10^T^; - = inoculation with Brucella broth. Each challenge was 6 weeks apart.

2 K = Groups were necropsied 6 days after inoculation with either broth or *C. hepaticus*, except for Group 5, where birds were necropsied 6 weeks and 6 days after the single challenge.

On necropsy, SLD lesions on the liver were recorded, and bile samples were collected to determine recovery of *C. hepaticus* after each challenge. Spleen and jejunum samples were collected to measure cytokine and TJ gene responses in challenged chickens, respectively. Every three weeks, blood was collected to monitor anti-*Campylobacter hepaticus* antibody levels and cloacal swabs were collected to determine the presence of *C. hepaticus.*

### DNA extractions

DNA from cloacal swabs, caecal contents, and bile samples were prepared using the DNeasy PowerSoil Kit (QIAGEN, Aarhus, Denmark) according to the manufacturer’s instructions. Upon collection, cloacal swabs were resuspended in 150 µL of nuclease-free water and stored at −20°C until DNA was extracted. Total DNA quality and concentrations was measured using NanoDrop and Qubit™ Fluorometric Quantitation (ThermoFisher, Waltham, MA) respectively. Aliquots were stored at −20°C until further use.

### Polymerase chain reaction (PCR)

Isolated DNA from cloacal swabs was subjected to PCR amplification to detect the presence of *C. hepaticus* DNA. PCR primers specific to *C. hepaticus* and *C. bilis* were used as previously described by Van et al. (2017b). End point PCR was carried out in a final volume of 20 μL using MyTaq™ 2x Master Mix (Meridian Bioscience, London, UK), primers at a final concentration of 250 nM each and 1 μL of template DNA. An Eppendorf Mastercycler Pro PCR instrument (Eppendorf, Hamburg, Germany) was used for amplification with cycling conditions of; 95°C for 1 min, 35 cycles of 95°C for 30 s; 56°C for 30 s, 72°C for 10 s and final extension for 72°C for 5 min. PCR with universal primers as used by Van et al. (2017a), targeting conserved bacterial 16S rRNA gene sequences was carried out as a positive control for PCR to demonstrate appropriate quality of all DNA templates.

### Recovery, identification and quantitation of *C. hepaticus*

To isolate *C. hepaticus,* 20 µL of bile samples were directly streaked onto HBA plates (Van et al., 2016). Remaining samples were stored at −20°C until DNA extraction. The identity of *C. hepaticus-*like colonies was confirmed by matrix assisted laser desorption/ionization time of flight mass spectrometry (MALDI-TOF MS) using a Microflex LT mass spectrometer (Bruker MALDI Biotyper System, Bruker Daltonics, Karlsruhe, Germany) according to the manufacturer's instructions. Quantitation of *C. hepaticus* in bile was performed using qPCR as described by Van et al. (2017b) in a total of 10 μL in triplicates. DNA derived from a culture of a known CFU/mL of *C. hepaticus* strain HV10^T^ was serially diluted 10-fold and used to generate a standard curve to determine efficiency of the reaction.

#### Enzyme-linked immunosorbent assay (ELISA)

Serum samples were collected every 3 weeks to monitor *C. hepaticus* specific antibodies over the course of the trial. Samples were centrifuged at 2000*xg* for 30 min and stored at −20°C. Parameters for optimising the ELISA assay are detailed by Muralidharan et al. (2020). Briefly, *C. hepaticus-*specific antibodies were measured by coating wells with *C. hepaticus* total protein extracts (TPE) diluted 1:100 to OD_600_=0.01 as the antigen. Non-specific binding sites were blocked using 5 % skim milk in phosphate buffered saline (PBS) + 0.05 % Tween 20 (PBST). Any antibodies raised against *C. jejuni* proteins, which could be cross-reactive to *C. hepaticus* proteins were depleted by pre-absorbing sera dilutions with *C. jejuni.* TPE diluted to OD_600_=0.1. Primary chicken sera were diluted 1:1000 and probed with goat anti-chicken secondary antibody diluted 1:2000. All wash steps were done using PBST. Novex^TM^ HRP Chromogenic Substrate (Invitrogen, Carlsbad, CA) was added and absorbances were measured using the POLARStar Omega Plate reader (BMG Labtech, Offenburg, Germany) at 652 nm. All serum samples were tested in triplicate. The positive controls used in the assay were sera from birds with known anti-*C. hepaticus* antibodies, as determined in our previous study (Muralidharan et al., 2020).

### Gene expression study

Spleen and jejunum samples were collected in 5 mL of RNA later™ (Invitrogen, Carlsbad, CA) for RNA extraction to measure tight junction protein and cytokine gene expression. Samples were stored at −20°C until used for extraction. Total RNA was extracted using the Maxwell® RSC simply RNA Tissue Kit (Promega, Madison, WI) as per the manufacturer’s instructions and reverse transcribed to cDNA using SuperScript™ IV First Strand Synthesis System (ThermoFisher, Waltham, MA). Quantitative PCR was performed using 2x KAPA SYBR® FAST mastermix (Roche, Basel, Switzerland) on the CFX Connect Real-Time System (Bio-Rad, Berkeley, CA) with the following temperature cycling conditions: 95°C for 3 min, 39 cycles of 95°C for 10 s; 60°C for 30 s and 72°C for 30 s. Gene expression was normalized against two reference genes, GAPDH and β-actin, using the formula:

$Relative\;gene\;expression=\frac{{\left(E_{GOI}\right)}^{\Delta Ct\;GOI}}{GeoMean[{\left(E_{REF}\right)}^{\Delta Ct\;REF}]}\;$. Expression of the target genes were analysed using the primers detailed in Table 2.

**Table 2:** Primer details for qPCR.

Gene	Primer sequence (5′>3′)	Size of Amplicon	Accession Number	References
GAPDH	F: CCTAGGATACACAGAGGACCAGGTT R: GGTGGAGGAATGGCTGTCA	64	NM_204460.1	(He et al., 2019)
β-actin	F: CCAGACATCAGGGTGTGATGG R: CTCCATATCATCCCAGTTGGTGA	137	AJ719605	(Borowska et al., 2016)
ZO1	F: GGAGTACGAGCAGTCAACATAC R: GAGGCGCACGATCTTCATAA	101	XM_413773	(Emami et al., 2019)
ZO2	F: GCGTCCCATCCTGAGAAATAC R: CTTGTTCACTCCCTTCCTCTTC	89	NM_204918	(Emami et al., 2019)
CLDN5	F: GCAGGTCGCCAGAGATACAG R: CCACGAAGCCTCTCATAGCC	162	NM_204201	(Nguyen et al., 2021)
IL-6	F: GCGAGAACAGCATGGAGATG R: GTAGGTCTGAAAGGCGAACAG	143	NM_204628	(Jiang et al., 2011)
IL-1β	F: GAAGTGCTTCGTGCTGGAGT R: ACTGGCATCTGCCCAGTTC	86	NM_204524.1	(Crhanova et al., 2011)

### cDNA library construction, sequencing and quality control for RNA sequencing

The following samples were used for RNA sequencing: Unchallenged (Group 2): birds Or32 and Or44; Single challenge (Group 1): birds Or87, Or68 and Or45; Triple challenge (Group 7): birds Ye53, Or42 and Wh49. An Agilent Bioanalyzer 2100 system (Agilent Technologies, Santa Clara, CA) was used to check the quality of the RNA. Sequencing was conducted by the Australian Genome Research Facility (AGRF) using an Illumina NovaSeq X Plus platform (Illumina, Inc., San Diego, CA) and 150-bp paired-end reads were generated. Image analysis was performed in real time by the NovaSeq Control Software (NCS) v1.2.0.28691 and Real Time Analysis (RTA) v4.6.7 (Illumina, Inc., San Diego, CA), running on the instrument computer. The Illumina DRAGEN BCL Convert 07.021.645.4.0.3 pipeline was used to generate the sequence data (Illumina, Inc., San Diego, CA). Quality of Illumina reads were checked using FastQC software (Galaxy version 24.1.3) to assess for the quality of the reads.

### Mapping reads, quantification of gene expression levels and differential expression analysis

Differential expression analysis was also performed on ExpressAnalyst using the DESeq2 package available at https://www.expressanalyst.ca/. Genes with a P value < 0.05 and a fold change of ≥1 were considered to be significantly differentially expressed and were selected for further investigation. Gene ontology and PANTHER enrichment analysis of differentially expressed genes (DEGs) was also conducted using the ExpressAnalyst platform.

### Statistical analysis

All statistical analysis was carried out using GraphPad Prism (9.3.1) software (San Diego, CA). *P-*values were calculated at 95 % confidence interval. Significance of *C. hepaticus* numbers in bile we calculated using unpaired t-test using Welch’s correction. Correlation of spleen and body weights was determined using Pearson, with a 95 % confidence level. The variance in antibody levels between each infection group through the trial was determined using unpaired t-test and one-way ANOVA with the Geisser-Greenhouse correction used when *P* *=* 0.05. qPCR data was analysed using unpaired t-test with Welch’s correction. For groups which were not normally distributed, a Mann Whitney’s t-test was implemented.

## Results

### SLD reinfection protects chickens from liver lesion development

The clinical presentation of SLD, along with the prevalence and recovery of *C. hepaticus* from the bile is shown in Table 3. Most birds in the positive control groups (Group 1, 6 and 10) developed typical liver lesions, ranging in number from 10 to 1000+ miliary liver lesions. As expected, no liver lesions were observed in any of the negative control groups (Group 2, 4 and 8). After two challenges, three birds from Group 3 presented with less than five lesions, and no lesions were observed for other birds. For Groups 9 and 7, which were challenged two and three times respectively, no liver lesions were observed at necropsy.

**Table 3:** The prevalence of *C. hepaticus* from chickens after one, two or three challenges over 12 weeks.

	Group 1^2^	Group 2^3^	Group 3	Group 4	Group 5	Group 6	Group 7	Group 8	Group 9	Group 10
No. of challenges	1	0	2	0	1 (necropsied after 6 weeks)	1	3	0	2 (12 weeks apart)	1
Liver lesions/number of birds	10-500/12 birds	0	1-5/3 birds	0	0	5-1000+/10 birds	0	0	0	50-1000+/10 birds
Cultured from bile	N/A^1^	N/A	10/12	0/12	11/12	8/12	11/12	0/12	12/12	11/12
Cloacal swab PCR positive out of 12 birds after 6 weeks/9 weeks	N/A	N/A	N/A	N/A	7/N/A	N/A	6/5	N/A	7/2	N/A
Quantification from bile via qPCR (CFU/ml)	4.64 × 10^4^ to 5.9 × 10^8^	N/A	N/A	N/A	2.2 × 10^2^ to 2.54 × 10^4^ (*P* < 0.0001)*	N/A	N/A	N/A	(3.16 × 10^3^ to 6.57 × 10^8^ (*P* < 0.0001)^	N/A

1 N/*A*= methodology was not performed for that group. ^2-3^ For Group 1 and 2, recovery of *C. hepaticus* was not performed via the plating method, however, was done by qPCR only.

⁎ Statistically significant compared to a single *C. hepaticus* infection (Group 1).

^ statistically significant compared to a single long-term *C. hepaticus* infection.

### *C. hepaticus* numbers in bile decreased over time but increased again following reinfection

Interestingly, except for the negative control groups, *C. hepaticus* was isolated from bile samples from all groups (Table 3). This indicates that although *C. hepaticus* was still present in bile, the birds did not develop lesions upon re-exposure. PCR of the cloacal swab samples showed the number of birds colonised with *C. hepaticus* reduced over time. In Group 9, at six weeks post infection, more than 50 % (7 out of 12) birds had detectable *C. hepaticus* DNA in cloacal swabs whereas at nine weeks post infection, only 2 out of 12 birds were positive (17 %). For birds challenged three consecutive times (Group 7), only 6 birds had detectable *C. hepaticus* in the swabs 6 weeks after the first challenge and 5 birds were positive three weeks after the second challenge, however, *C. hepaticus* could be cultured from the bile of 11 birds 6 days after the third challenge.

**Fig. 1:**
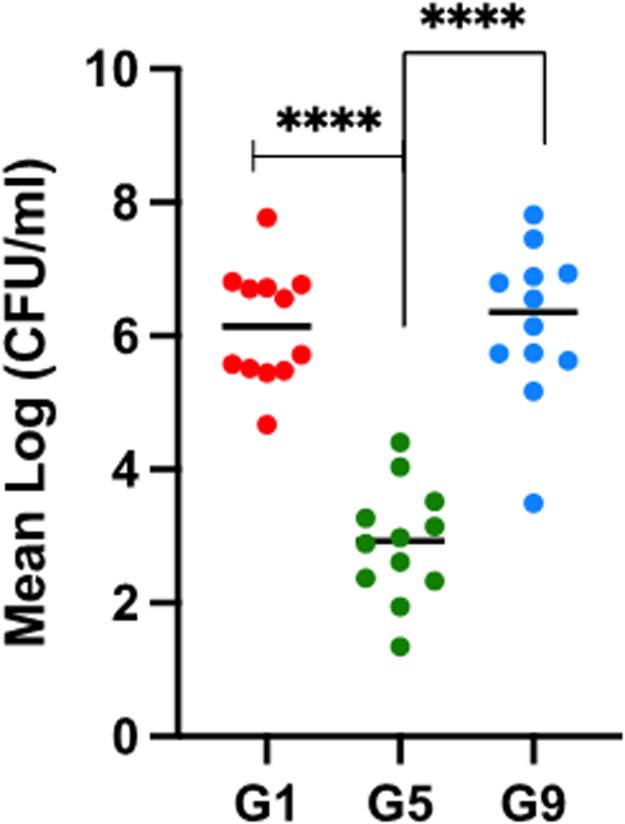
Quantification of *C. hepaticus* in bile using qPCR. CFU/ml are represented as a mean where, *n* = 3 over 2 independent experiments. Unpaired t-test using Welch’s correction was used when *P=* 0.05 was used to measure statistical significance. ****: *P* < 0.0001.

Quantitative PCR was used to enumerate *C. hepaticus* in bile samples (Table 3 and Fig. 1). For Group 1 (positive control), *C. hepaticus* was recovered from the bile of all birds six days after the initial challenge, ranging from 4.6 × 10^4^ to 5.9 × 10^8^ CFU/ml. Six weeks after the first challenge, a significant decrease in *C. hepaticus* counts in bile was observed for all 12 birds in Group 5 (2.2 × 10^2^ to 2.5 × 10^4^ CFU/ml) (*P* < 0.0001). All birds had low growth of *C. hepaticus* on isolation plates compared to the recovery observed in all positive groups. In Group 9, a second challenge occurred 12 weeks after the first exposure. Six days after the second exposure, *C. hepaticus* numbers significantly increased for all birds in Group 9 (3.2 × 10^3^ to 6.6 × 10^8^ CFU/ml) (*P* < 0.0001) compared to six weeks after a single exposure for Group 5. Overall, *C. hepaticus* numbers in bile decreased over time but significantly increased again following reinfection.

### Anti-*C. hepaticus* serum IgY antibody levels peaked six weeks post-infection but then declined after nine weeks

**Fig. 2:**
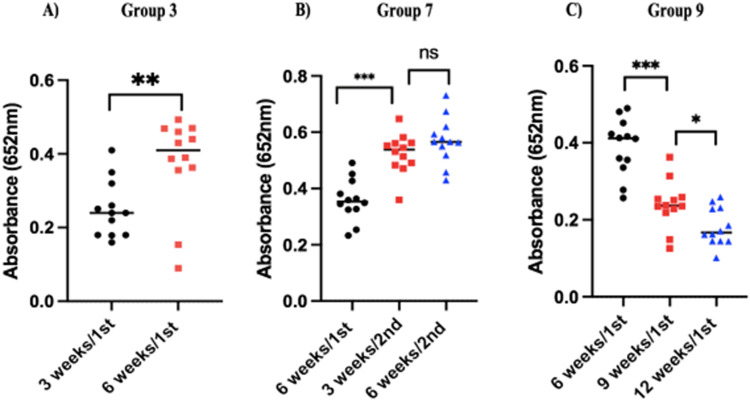
Anti-*C. hepaticus* IgY antibody levels in serum. (A) single challenge (Group 3), (B) three consecutive challenges (Group 7) and (C) two non-consecutive challenges (Group 9). Absorbances were measured in triplicates and mean calculated. For each group, unpaired t-test and One-way ANOVA with the Geisser-Greenhouse correction was used when *P=* 0.05.

Serum samples were collected every 3 weeks to monitor *C. hepaticus-*specific IgY antibody levels throughout the rechallenge periods using the SLD ELISA1 assay (Muralidharan et al., 2020). Absorbance readings over 0.1 were considered positive. The assay was not conducted for Group 1 because six days after a single challenge was not sufficient time for IgY to rise to a detectable level. After a single challenge, antibody levels differ significantly between 3 and 6 weeks (*P* = 0.0057) for Group 3. The mean absorbance increased from 0.25 to 0.37. Generally, IgY levels increased for most birds after 6 weeks (Fig. 2A). A similar pattern was observed after three consecutive reinfections (Group 7). The mean absorbance increased from 0.36 to 0.52 (*P* *=* 0.0001) three weeks after the second reinfection. Six weeks after the secondary infection, the mean plateaued to 0.57 (not statistically significant when compared to 3 weeks post challenge) (Fig. 2B).

As can be seen in Group 9, 12 weeks after a single infection, there was a significant reduction in antibody levels over time. At 6 weeks after a first infection, absorbances averaged 0.42 and decreased to 0.24 (*P* *=* 0.001) and 0.19 (*P* *<* 0.0001) at 9 and 12 weeks, respectively (Fig. 2C).

### SLD re-exposure led to increased expression of tight junction protein genes from jejunum samples

**Fig. 3:**
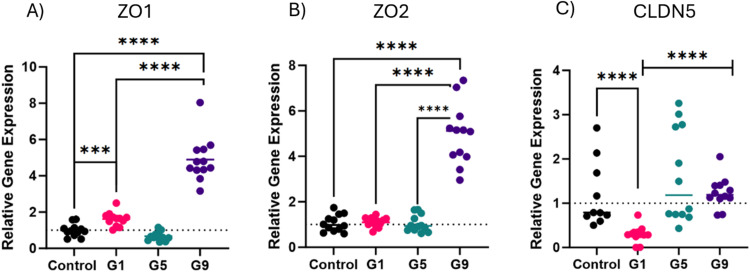
Changes in gene expression of tight junction protein genes from jejunum samples. Gene expressions were studied 6 days after single challenge in G1, 7 weeks after a single challenge in Group 5 and 6 days after the second challenge in G9. Gene expression was normalised using ß-actin and GAPDH as the reference genes (represented as the dotted line for each graph). The relative gene expression changes were calculated using the geometric mean for each reference gene. Fold change is represented as mean ± SD where *n* = 12 birds per group, assayed in triplicate. Unpaired t-test using Welch’s correction was used at *P=* 0.05 was used to measure statistical significance. ***: *P* ≤ 0.001; ****: *P* < 0.0001. For groups which were not normally distributed, a Mann Whitney’s t-test was implemented.

The relative gene expression of three tight junction’s proteins were measured to understand barrier support and integrity during *C. hepaticus* infection. The expression of ZO1 significantly increased six days after a single infection (*P* = 0.0005) (Group 1). No significant difference in expression of ZO2 was observed for Group 1. Six weeks after a single challenge, ZO1 levels decreased, although not significantly (Group 5). ZO2 levels remained unchanged. Interestingly, a secondary challenge significantly raised both zonula occluden gene transcription levels compared to a single challenge. The mean fold change was 4.89 (*P* < 0.0001) and 4.94 (*P* < 0.0001) for ZO1 and ZO2, respectively (Fig. 3A and 3B).

After a single challenge (Group 1), CLDN5 levels significantly decreased, with the mean decreasing to a relative expression level of 0.3 (*P* < 0.0001) compared to the uninfected control group. CLDN5 levels increased to a relative expression level of 1.6; 6 weeks and 6 days after challenge (Group 5) (*P* < 0.0001). Some variation in CLDN5 expression in Group 5 was observed, whereby 6 birds ranged between 1.5-3.3-fold changes. The remaining 6 birds had similar transcription levels to birds in Group 1. Secondary *C. hepaticus* challenge (Group 9) resulted in a significantly higher expression of CLDN5 compared to a single challenge (*P* < 0.0001). No statistically significant difference in CLDN5 expression was observed between Group 9 and Group 5 (Fig. 3C).

### SLD induces significant inflammatory responses in infected chickens

**Fig. 4:**
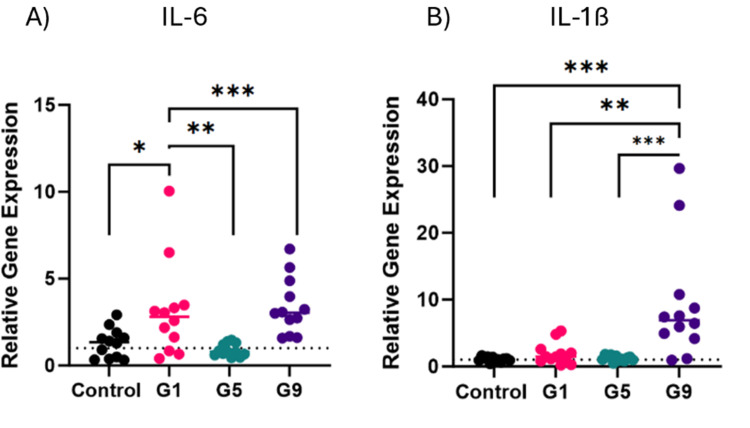
Changes in expression of pro-inflammatory cytokines IL-6 and IL-1ß genes in spleen samples. Gene expression was studied 6 days after a single challenge in Group 1, 7 weeks after a single challenge in Group 5, and 6 days after the second challenge in Group 9. Gene expression was normalised using ß-actin and GAPDH as the reference genes (represented as the dotted line for each graph). The relative gene expression changes were calculated using the geometric mean for each reference gene. Fold change is represented as mean ± SD where *n* = 12 birds per group, assayed in triplicate. Unpaired t-test using Welch’s correction was used at *P=* 0.05 was used to measure statistical significance. *: *P* ≤ 0.05; **: *P* ≤ 0.01; ***: *P* ≤ 0.001. For groups which were not normally distributed, a Mann Whitney’s t-test was implemented.

The expression of interleukins IL-6 and IL-1β in spleen samples of the negative control group (G2), positive control group (G1), the group necropsied 6 weeks and 6 days after a single exposure (G5); and the group that received two challenges 12 weeks apart and necropsied and sampled 6 days after the second challenge (G9), were measured by qRT-PCR to characterise the pro-inflammatory responses following infection. The expression of IL-6 was significantly elevated six days after a single challenge (*P* = 0.0173), with a mean of fold change of 3.2. However, there was no significant change in IL-1β expression after a single challenge. Seven weeks after the single challenge, a significant reduction in IL-6 levels was observed in Group 5 with a relative mean of 0.9 (*P* = 0.0068) (Fig. 4A).

Both pro-inflammatory cytokine levels increased after a second challenge in Group 9, with mean fold changes of 9.3 (*P* = 0.0005) and 3.4 (*P* *=* 0.0002) for IL-1β and IL-6, respectively compared to Group 5 (Fig. 4A and 4B).

To further analyse the immune responses of birds to *C. hepaticus* infection, eight libraries were sequenced from RNA extracted from samples of eight individual birds (two negative controls, three singly challenged and three triply challenged birds). A total of 76.69 Gb of data was obtained. The mapping rate of the sequences to the reference transcriptome exceeded 75 %, measuring the transcription of 15,087 genes.

**Fig. 5:**
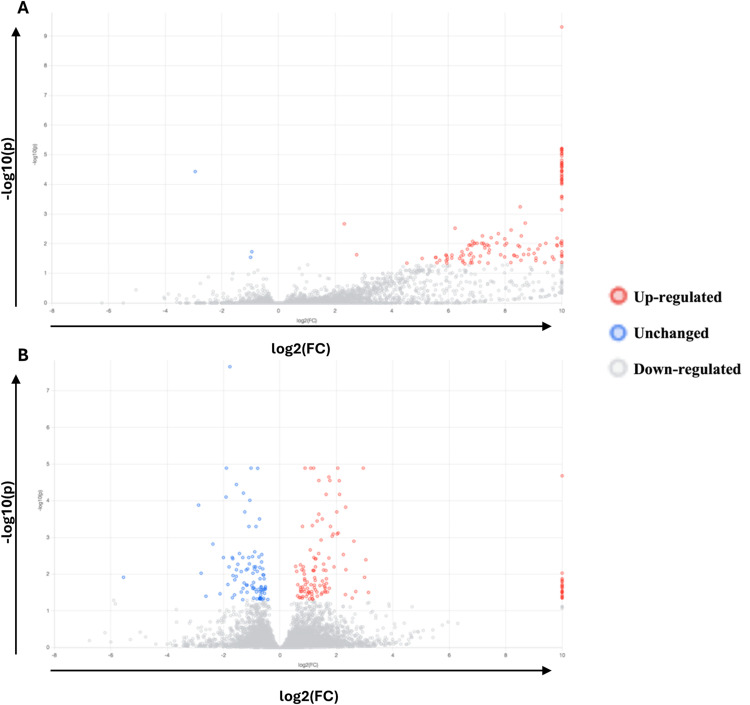
Volcano plot of differentially expressed genes (DEGs) in RNA-Seq. (A) Volcano plot of DEGs in RNA-seq of a single *C. hepaticus* challenge versus control (unchallenged). (B) DEGs in RNA-seq of a triple *C. hepaticus* challenge versus control.

Comparison of the expression levels between infection statuses, 139 differentially expressed genes (DEGs) were obtained for a single challenge (Fig. 5A) and 220 DEGs for a triple challenge when compared to the control (Fig. 5B). Three hundred and twenty-three DEGs were identified when comparing a triple challenge to a single challenge. Table 4 outlines the key pathways of interest identified during enrichment analysis. For a single challenge compared to the control, the majority of DEGs related to metallopeptidase activity, hormone secretion/activity, leukocyte differentiation, apoptotic process, sensory perception to pain and humoral immune response were up regulated. During a triple challenge, the majority of enriched DEGs are associated with cell adhesion, angiogenesis, protein kinase inhibitor activity, and blood coagulation. When comparing a triple to a single challenge, many DEGs were significantly downregulated in enrichment pathways including cytokine activity, JAK STAT cascade, leukocyte differentiation, and positive regulation of immune system. DEGs within the response to toxin pathway was only significant for a triple versus single challenge, but not significantly differentiated when compared to the untreated group.

**Table 4:** Results of GO and PANTHER enrichment analysis of differentially expressed genes (DEGs) using the Express Analyst platform.

Group	Category^1,2^	Term	*P-*value^3^	Genes^4,5^
**DEGs for single challenge versus control**	GO_BP	Hormone secretion/activity	0.0147	*HNF4A ^(+8.2)^, APOA4 ^(+10.0)^, GIP ^(+10.0)^, IAPP ^(+10.0)^, CCK ^(+10.0)^, UCN3 ^(+10.0)^*
	GO_BP	Leukocyte differentiation	0.0192	*SI ^(+7.3)^, LCT ^(+5.6)^, HHIP ^(+9.9)^, HKDC1 ^(+10.0)^*
	GO_BP	Cytokine mediated signalling pathway	0.0292	*KRT40 ^(+7.7)^*
	GO_BP	Regulation of T cell activation	0.0292	*CLDN3 ^(+8.5)^*
	GO_BP	Humoral immune response	0.037	*VILI ^(+6.8)^, PIGR ^(+9.2)^*
	GO_BP	Bile acid metabolic process	0.0481	*SLC4A4 ^(+6.4)^*
	GO_BP	Cytokine metabolic process	0.0481	*HNF1A ^(+7.4)^*
	PANTHER_BP	Sensory perception of pain	0.0229	*GIP ^(+10.0)^, IAPP ^(+10.0)^*
	PANTHER_BP	Response to interferon gamma	0.0481	*IFITM3 ^(+9.9)^*
	PANTHER_BP	Apoptotic process	0.0383	*VILI ^(+7.8)^, PRUNE2 ^(+6.1)^, CIDEC ^(+7.8)^, DPEPI ^(+6.5)^*
	PANTHER_MF	Metallopeptidase activity	1.53E-04	*MEP1B ^(+6.1)^, ADAM9L ^(+8.6)^, MEP1A ^(+6.9)^, ACE ^(+8.7)^, ACE2 ^(+2.3)^, DPEP1 ^(+7.5)^*
**DEGs for triple challenge versus control**	GO_BP	Response to temperature stimulus	0.0121	*ADGRF5 ^(+10.0)^, HNF4A ^(+1.6)^*
	GO_BP	Lipid catabolic process	0.038	*HNF4A ^(+10.0)^, HNF4G ^(+10.0)^*
	GO_MF	Protein kinase inhibitor activity	9.78E-3	*GH ^(+1.3)^, CCK ^(+10.0)^, IAPP ^(+10.0)^, SST ^(+10.0)^*
	PANTHER_BP	Blood coagulation	0.0224	*VWF ^(+0.8)^, HNF4A ^(+10.0)^, TFP12 ^(−1.1)^*
	PANTHER_BP	Angiogenesis	0.0923	*DLL4 ^(−2.0)^, ANXA2 ^(−0.8)^, NUS1 ^(−1.3)^, XBP1 ^(+1.6)^*
	PANTHER_BP	Cell adhesion	0.0748	*CDH23 ^(−1.8)^, DCHS1 ^(+1.7)^, CTNNA2 ^(−1.5)^, VWF ^(+0.8)^, FN1 ^(+1.4)^, VCAN ^(+2.9)^*
**DEGs for triple challenge versus single challenge**	GO_BP	Positive regulation of immune system	6.93E-4	*LEPR ^(+2.9)^, ADGRF5 ^(−6.7)^, KL ^(+1.5)^*
	GO_BP	Leukocyte differentiation	4.26E-4	*B3GAT1 ^(+2.3)^, SI ^(−1.8)^, HKDC1 ^(−3.4)^, LCT ^(−6.7)^, HHIP ^(−6.6)^, SLC3A1 ^(−5.6)^, KL ^(−3.0)^, HEXA ^(−8.7)^, KLB ^(+10.0)^, GPDIL2 ^(−5.1)^, GK2 ^(−4.3)^*
	GO_BP	Regulation of T cell activation	5.14E-3	*CLDN3 ^(−8.0)^, CLDN2 ^(+10.0)^*
	GO_BP	Immune response	8.85E-3	*FLT3 ^(−6.1)^, EPHA10 ^(+2.4)^, EPHA7 ^(−4.8)^, PDGRFB ^(+10.0)^, BMX ^(+10.0)^, FRK ^(+5.9)^, EPHA1 ^(+2.3)^, STYK1 ^(+1.4)^*
	GO_BP	Response to toxin	0.019	*SLC8A3 ^(+2.1)^, SLC39A10 ^(−5.3)^, ATP7B ^(+1.6)^*
	GO_BP	Positive regulation of T cell proliferation	0.042	*FFAR4 ^(−5.8)^*
	GO_BP	JAK STAT cascade	0.0424	*COCH ^(−4.5)^, RDX ^(+0.9)^, VILI ^(+4.2)^, WIPF3 ^(−6.7)^, F2 ^(−3.9)^, EPS8 ^(−5.5)^*
	GO_MF	Cytokine activity	0.0017	*NPC1 ^(+10.0)^, APOB ^(−2.31)^, ATP8B1 ^(+0.9)^, APOA1 ^(−4.8)^*

1,2 MF denotes molecular function for each database; BP denotes biological process.

3 statistically significant DEG’s at *P* = 0.05.

3,4 (+) indicates an up-regulated DEG; (-) indicates a down-regulated DEG.

Changes occurred between the challenge statuses for multiple immune response and barrier integrity/function DEGs (Table 5). Six days after a first *C. hepaticus* challenge, all birds show an upregulation in pro- inflammatory chemokines including IL-6, IL-1B, IL-8 and IL-17A. The greatest fold change was observed for pro-inflammatory cytokine, IL-17A. At the same time, the expression of the anti-inflammatory cytokine IL-10 also increased. While birds demonstrated a downregulation of CLDN5 and MUC1; tight junction proteins, MUC4 and MUC13 were moderately upregulated. Many pro- inflammatory interleukins were downregulated following a triple challenge compared to a single challenge, suggesting a negative regulation of the immune response.

**Table 5:** Fold changes of differentially expressed genes (DEG) specifically related to cytokine production/regulation/function, immune responses including inflammatory responses and adaptive immunity; and genes related to barrier support and integrity of the intestinal tract.

DEGs	Single challenge vs control^2^	Triple challenge vs control^2^	Triple vs single challenge^2^
*Innate and adaptive immunity*			
Interleukin 6 (IL-6)	**1.48**	**2.74**	**N/A**^1^
Interleukin-1B (IL-1B)	**1.60**	**0.67**	**−0.16**
Interleukin 8 (IL-8)	**1.26**	**N/A**	**N/A**
Interleukin 10 (IL-10)	**1.23**	**2.28**	**0.89**
Interleukin 17A (IL-17A)	**4.51**	**1.68**	**−3.70**
*Barrier support*			
Claudin 5 (CLDN5)	**−0.40**	**1.77**	**1.70**
Tight Junction Protein 1 (TJP1)	**1.76**	**1.91**	**1.57**
Tight Junction Protein 2 (TJP2)	**2.03**	**2.52**	**N/A**
Mucin 13 (MUC13)	**1.87**	**3.46**	**3.86**
Mucin 1 (MUC1)	**−0.54**	**3.16**	**2.29**
Mucin 4 (MUC4)	**1.28**	**2.47**	**1.19**
Fatty acid binding protein 1 (FABP1)	**2.78**	**9.8**	**8.61**

1 N/A; DEGs were not annotated and fold changes not reported for the group comparison.

2 For each reinfection status comparison (columns), numerical values denote statistically significant fold changes of DEGs measured at *P=* 0.05.

Comparatively, all barrier support DEGs were more highly transcribed after a triple challenge compared to a single, suggesting potential intestinal barrier repair and clearance of *C. hepaticus*. The expression of FABP1 (Fatty Acid Binding Protein 1), which is primarily involved in the transport and metabolism of fatty acids within cells, particularly in the liver, showed an 8.6-fold change in the triple version single challenge.

## Discussion

Our study shows that layers infected with *C. hepaticus* can develop immunity that prevents the manifestation of liver lesions following repeated exposure to the pathogen. We assume they are a good proxy/indicator of other clinical manifestations such as drop in egg production. Study of egg production was beyond the scope of this experiment as much larger groups would be required to generate statistically significant results.

Six weeks after a single infection challenge, over 50 % of birds had detectable levels of *C. hepaticus* in cloacal swab samples. This percentage decreased to 17 % of birds 9 weeks after challenge. Over time, most birds can clear a *C. hepaticus* infection. Physical factors of the GI tract including pH, level of mucins, bile, motility and composition of resident microbiota are likely to affect the pathogen’s ability to colonise. *C. hepaticus* was recovered from bile in most birds across all infection statuses. However, comparisons with its presence in the GI tract are limited, as cloacal swabs were sampled pre-challenge, while bile was collected six days post-challenge. Here we demonstrated that *C. hepaticus* numbers reduced two thousand-fold seven weeks after a single infection, peaking at 6.2 × 10^6^ CFU/ml after a second challenge. Since bile is a challenging environment for survival, it has been demonstrated that *C. hepaticus* upregulates a range of genes associated with stress tolerance, glucose utilization, hydrogen metabolism and sialic acid biosynthesis. This allows *C. hepaticus* to adapt to the oxygen-limiting and high copper environment (Van et al., 2017c). No liver lesions were detected in the reinfection groups 7 and 9, while only 1-5 lesions were detected in 3 out of 12 birds in Group 3. However, 10-12 out of 12 of the birds in each group harboured *C. hepaticus* in the livers. This demonstrates that the birds can recover from infection, most likely due to an immune response that protects the liver but does not clear the bacteria from the gall bladder. Furthermore, its presence in the bile does not always lead to miliary lesions on the liver.

The effect of *C. hepaticus* infection on adaptive immunity was assessed by measuring the IgY levels*.* It was observed that anti-*C. hepaticus* IgY titres increased 3-6 weeks post first challenge. This is in some agreement with studies reporting that specific IgY levels started to increase within 2-3 weeks during *C. jejuni* infection (Cawthraw et al., 1998; Han et al., 2016; Pielsticker et al., 2016; Mortada et al., 2021). Detectable levels of antibodies dropped 12 weeks post single infection (but were still considered anti-*C. hepaticus* positive). It has been shown that *C. jejuni* intestinal colonisation does not significantly affect adaptive immunity but dissemination of *C. jejuni* to the liver triggers Th1 and Th2 responses, resulting in a spike of specific IgY antibodies which in turn increases hepatic clearance of the pathogen (Chagneau et al., 2023). This may explain why we observed a continued rise in IgY levels six weeks after a single infection. Upon a secondary challenge, no liver lesions were observed, suggesting the presence of memory immune cells circulating in the blood stream, along with some remaining antibody responses were sufficient to provide protection against lesion development. The results also suggest that the recurrence of SLD in flocks over a short period may occur because not all birds are infected during the initial exposure, leaving them vulnerable to later infections.

IL-10 promotes the Th2 response, primarily through supressing pro-inflammatory cytokines or inhibiting the Th1 response (Sanjabi et al., 2009; Kogut and Arsenault, 2017). and is primarily produced by T-cells, B-cells and monocytes/M2 macrophages. Upon single *C. hepaticus* challenge, the expression of IL-10 increased to suppress inflammation, however, the expression of pro-inflammatory cytokines IL-6, IL-1β, IL-8, and IL-17A also increased to drive the inflammatory response to eliminate pathogens. This suggests a complex regulation of immune responses where IL-10 moderates’ inflammation to prevent tissue damage while allowing sufficient pro-inflammatory activity to combat the infection. The expression of pro-inflammatory cytokines was downregulated in the triple compared to single infection challenge, demonstrating a balanced immune response had already developed. This hypothesis is supported by the observed enriched pathways. In a single challenge, genes linked to cell death, pain perception, and immune response were mostly upregulated. However, in a triple challenge compared to a single challenge, many genes involved in immune activities, like cytokine production, the JAK-STAT cascade (a pathway crucial for controlling immune responses, cell growth, and differentiation), and immune cell development, were significantly downregulated. The difference in gene expression indicates a complex immune modulation in triple infections, suggesting a potentially adaptive mechanism at play.

Various factors can affect the permeability of the intestinal tight junction (TJ) barrier including commensal microbiota, luminal endotoxin, and pathogens. These factors can cross the epithelium and enter the circulation when the host experiences stress and inflammation (Shen et al., 2011). The upregulated expression of ZO1 and ZO2 genes after a second challenge may be attributed to inflammatory responses occurring in the gut. Pro-inflammatory cytokines mediate tight junction protein (TJP) regulation using myosin light chain kinase (MLCK) and damages the tight junction scaffolding by interfering with interactions between TJP and the actin-myosin cytoskeleton (Capaldo and Nusrat, 2009). The elevated expression of ZO1 and ZO2 could counteract the damage exerted by these cytokines.

Claudins are mainly responsible for linking adjacent enterocytes through interactions of their extracellular loops in TJ complexes. Specifically, CLDN5 is known to form tight junctions and decrease permeability. Interleukins such as IL-6 have been shown to affect the expression of other claudins such as CLDN2 (Suzuki et al., 2011) to counteract the inflammatory response in the chicken gut. An increase in CLDN5 expression 6 weeks after infection, indicates a recovery of the gut, primarily restoring pore formation and stability. A downregulation of the barrier forming CLDN5 from the first infection could indicate a disruption in the structure and permeability of TJs allowing *C. hepaticus* translocation into the liver and bile (Moore et al., 2019).

Mucin 1 (MUC1) is a cell surface mucin and to access its receptor, a pathogen must penetrate the protective mucus layer, comprising of secretory mucins such as mucin 2 (MUC2) and has been shown to be a major modulator of pathogen-induced inflammation (McAuley et al., 2007; Guang et al., 2012; Ng et al., 2016). Decreased expression of MUC1, observed following a single challenge, may contribute to higher inflammation levels. The observed elevation of inflammatory responses following a triple challenge compared to a single challenge demonstrated the enhanced protection of the birds against pathogens. Mucin 2 expression remained unchanged during all reinfections. It is secreted by goblet cells and forms a protective layer on the epithelial cells, defending the host against pathogenic bacteria (McGuckin et al., 2011). Unchanged mucin 2 levels may suggest unknown evasion mechanisms possessed by *C. hepaticus* which allows the pathogen to bypass the mucus barrier without directly affection its production.

Fatty acid binding protein (FABP1) plays a role in fatty acid metabolism and is recognised to be involved in both metabolic and inflammatory pathways (Chen et al., 2015; Cahyaningsih et al., 2018). The elevated expression of FABP1 during single and triple challenges could indicate birds’ response to liver stress. This suggests that FABP1 may help modulate the liver's reaction to infection-related stress.

In conclusion, this study showed that chickens can recover from SLD and then subsequently resist lesion formation following re-exposures to *C. hepaticus.* In response to these infections, the birds' immune system triggers a cascade involving the coordinated regulation of regulatory and pro-inflammatory cytokines, epithelial barrier components like mucin and tight junction proteins, and antibody responses. This process leads to a more balanced immune response during re-exposures compared to the first exposure, helping to prevent liver damage. These findings are crucial for creating effective management strategies in poultry farming, as they emphasise the potential to enhance disease resilience in flocks. Furthermore, the results suggest the potential for developing a vaccine for SLD and that a parenteral immune response may be sufficient to protect birds from this important disease.

## Declaration of competing interest

PCS, TBW and JH were employed by Scolexia Pty Ltd. The authors declare that the research was conducted in the absence of any commercial or financial relationships that could be construed as a potential conflict of interest.
